# Multicomponent Reactions between Heteroatom Compounds and Unsaturated Compounds in Radical Reactions

**DOI:** 10.3390/molecules28176356

**Published:** 2023-08-30

**Authors:** Akiya Ogawa, Yuki Yamamoto

**Affiliations:** 1 Organization for Research Promotion, Osaka Metropolitan University, 1-1 Gakuen-cho, Nakaku, Sakai, Osaka 599-8531, Japan; 2 Graduate Faculty of Interdisciplinary Research, University of Yamanashi, 4-4-37 Takeda, Kofu 400-8510, Japan; y.yamamoto@yamanashi.ac.jp

**Keywords:** heteroatom-centered radicals, interelement compounds, multicomponent addition, photoirradiation, radical cyclization, unsaturated compounds, eco-friendly process

## Abstract

In this mini-review, we present our concepts for designing multicomponent reactions with reference to a series of sequential radical reactions that we have developed. Radical reactions are well suited for the design of multicomponent reactions due to their high functional group tolerance and low solvent sensitivity. We have focused on the photolysis of interelement compounds with a heteroatom–heteroatom single bond, which readily generates heteroatom-centered radicals, and have studied the photoinduced radical addition of interelement compounds to unsaturated compounds. First, the background of multicomponent radical reactions is described, and basic concepts and methodology for the construction of multicomponent reactions are explained. Next, examples of multicomponent reactions involving two interelement compounds and one unsaturated compound are presented, as well as examples of multicomponent reactions involving one interelement compound and two unsaturated compounds. Furthermore, multicomponent reactions involving intramolecular cyclization processes are described.

## 1. Introduction

Currently, as the importance of environmentally friendly manufacturing practices increases, there is a strong need to reduce waste and improve elemental efficiency in organic synthesis. In particular, one-pot multicomponent reactions are expected to be an effective method for reducing the complexity of isolation operations in multistep reactions consisting of multiple components [1,2]. However, the suppression of side reactions among multicomponent substrates is an important issue in one-pot reactions of multicomponent reactions. In order to further increase atomic efficiency, it is expected to be effective to utilize addition-type reactions that can ideally incorporate all substrates into the product, rather than substitution-type reactions that involve the byproduction of leaving groups. Although acid/base catalysts and transition metal catalysts have been widely used in conventional addition reactions, these reactions are easily affected by solvents. In contrast, the utilization of radical reactions with high tolerance for a variety of solvents is expected to be effective in order to uniformly dissolve substrates of multiple components [3,4,5,6,7,8]. Furthermore, we have come to believe that radical addition reactions that can be induced by light are the most effective reaction systems for multicomponent reactions, avoiding the use of radical initiators whose residues are waste products [9,10,11,12,13,14,15]. In particular, light irradiation in the near-ultraviolet to visible region is considered preferable to suppress side reactions of unsaturated compounds such as polymerization.

Based on this idea, we have been studying multicomponent radical addition reactions to carbon–carbon and carbon–nitrogen unsaturated compounds by selecting inter-element compounds having absorption in the near-ultraviolet to visible regions. This mini-review deals with an overview of the following three categories of multicomponent radical reactions that we have studied: (1) a reaction using multicomponent interelement compounds to one unsaturated compound, (2) a reaction using multicomponent unsaturated compounds to one interelement compound, and (3) a multicomponent reaction incorporating cyclization reactions.

## 2. General Concept for Photoinduced Radical Addition of Interelement Compounds to Unsaturated Bonds

Interelement compounds with heteroatom–heteroatom single bonds, in which a solitary electron pair exists on the heteroatom, usually have absorption based on n→σ* transitions in the near-UV to visible region, homolysis of the interelement bond proceeds upon photoirradiation in this region, and the corresponding heteroatom-centered radicals are generated. When carbon–carbon unsaturated compounds coexist in this reaction system, the radical addition reaction of interelement compounds to unsaturated compounds is expected to proceed with the generated heteroatom-centered radicals as the key active species. This radical addition reaction consists of the following two-step reaction process: **step 1**: the formation of carbon radicals by the addition of heteroatom-centered radicals to carbon–carbon unsaturated bonds, and **step 2**: capturing the formed carbon radicals by an interelement compound. As an alternative pathway for **step 2**, the coupling reaction between the carbon radical and the heteroatom-centered radical can be considered. However, the concentration of heteroatom radicals in the reaction system is relatively very low because heteroatom radicals easily dimerize each other at a diffusion-controlled rate to regenerate the starting interelement compounds, and thus the radical coupling pathway is not the major reaction process. In **step 1**, low-period heteroatom radicals are more reactive than high-period heteroatom radicals of the same family, while in **step 2**, high-period interelement compounds have a higher ability to capture carbon radicals than low-period interelement compounds of the same family because the binding energy of the high-period interelement bonds is relatively smaller. Thus, if the low-period interelement compound is used alone for photoinduced radical addition to alkenes, the undesirable polymerization reaction would be expected to proceed in preference to the radical addition reaction due to the lower carbon-radical-capturing ability of the low-period interelement compound. In contrast, when high-period interelement compounds are used alone, the desired radical addition reaction is expected to be less likely to proceed due to the lower reactivity of the high-period heteroatom radicals. To solve these problems and to make the radical addition reaction highly selective, it is expected that a combination of high-period and low-period interelement compounds will be promising: namely, a highly reactive low-period heteroatom radical selectively attacks alkenes, and the carbon radicals generated by this attack are selectively trapped by high-period interelement compounds.


(1)

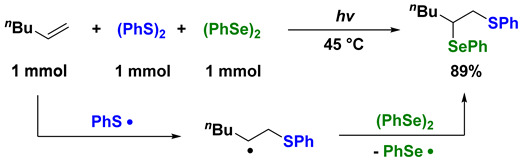



Taking disulfide and diselenide as examples to illustrate radical addition to 1-hexene, it is clear that when disulfide or diselenide is used alone, the radical addition reaction proceeds inefficiently: the 1,2-dithio- and 1,2-diseleno-hexenes were obtained in 7% and 9% yields, respectively. In the disulfide–diselenide binary system, however, the thioselenation product is obtained regioselectively in high yield (89% yield) [16]. This is because the highly reactive thiyl radical regioselectively attacks the terminal position of the alkene, and the generated carbon radical is selectively captured by the diselenide (Equation (1)). Based on this concept, we developed a series of radical addition reactions of interelement compounds to various unsaturated compounds such as alkynes, alkenes, allenes, conjugate dienes, isocyanides, etc. Some related works were successfully developed by other researchers [17,18,19,20,21,22,23,24,25,26].

When designing radical reactions of interelement compounds, it is necessary to consider not only the period but also the group. In the case of group 17 interelement compounds, which are molecular halogens, the resulting halogeno radicals have no substituent, and therefore, it is required to control the radical addition reaction by the properties of the element itself. Furthermore, halogeno radicals are highly reactive, and with alkenes, the hydrogen abstraction reaction at the allylic position of alkenes usually proceeds in preference to the desired radical addition reaction. In sharp contrast, group 16 interelement compounds, which are organic dichalcogenides such as (PhS)_2_, (PhSe)_2_, and (PhTe)_2_, can control the reaction by means of substituents. Since chalcogeno radicals such as PhS•, PhSe•, and PhTe• are less reactive than halogeno radicals, hydrogen abstraction at the allylic position usually does not occur, and the radical addition reaction proceeds preferentially. In addition, since chalcogeno radicals have only one substituent, the steric hindrance is not so important when the radical addition is performed. Organic dichalcogenides have their absorption in the near-ultraviolet to visible region based on the n→σ* transition and can generate the corresponding radical species by homolysis. In particular, organic diselenides and ditellurides can undergo homolysis upon sunlight irradiation.

Group 15 interelement compounds, which are organic dipnictogenides such as (Ph_2_P)_2_, have two substituents on each phosphorus group, making them susceptible to steric hindrance. In addition, trivalent species readily convert into pentavalent species upon exposure to air or moisture, so there are many factors that control the reaction. Furthermore, arsenic and antimony have problems such as high toxicity. Although (Ph_2_Bi)_2_ is a known compound, its synthesis and handling require specialized techniques.

Group 14 and 13 interelement compounds do not have lone pairs, so the homolysis of interelement bonds using the n→σ* transition cannot be used. In the case of group 14 interelement compounds, steric hindrance is a major problem due to the large number of substituents. In the case of group 13 interelement compounds, since there is an empty orbital, if homolysis is performed as it is, it becomes a 5-electron radical species, which is far away from the octet and becomes unstable. In addition, side reactions due to the coordination of Lewis bases to empty orbitals must be controlled.

Note that many second-period inter-element compounds with isolated electron pairs are unstable due to the short bonding distances of the interelement bonds and the large electronic repulsion between adjacent isolated electron pairs, and some peroxides and diazo compounds are explosive.

Keeping in mind the above considerations regarding the group and period, radical addition reactions to unsaturated bonds using mixed systems of multiple interelement compounds are discussed in the next section.

## 3. Photoinduced Radical Addition to Unsaturated Bonds Using Mixed Systems of Interelement Compounds

Addition reactions to terminal alkynes by binary systems of group 15–17 interelement compounds that have a lone pair of electrons and can generate heteroatom radicals upon irradiation with near-ultraviolet to visible light were investigated. In the case of group 16–17 binary systems, molecular halogens (X_2_) act as oxidizing agents for organic dichalcogenides (RCh-ChR) to generate the corresponding chalcogenyl halides (RChX), which are well-known to add to alkynes via an ionic process such as electrophilic addition.

**Scheme 1:**
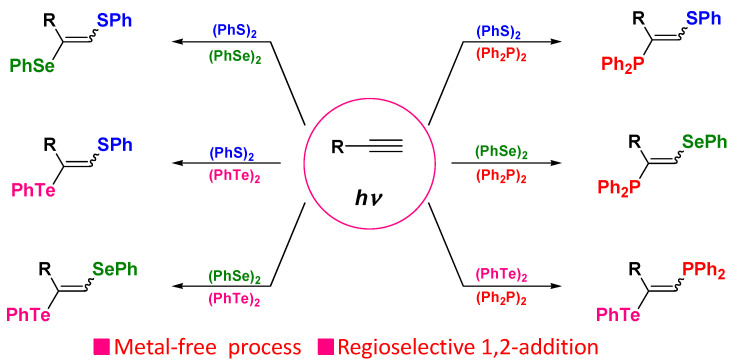
Simultaneous introduction of two different heteroatom groups into alkynes.

In the case of group 16 binary systems, photoinduced radical addition to terminal alkynes successfully occurs with (PhS)_2_-(PhSe)_2_, (PhS)_2_-(PhTe)_2_, and (PhSe)_2_-(PhTe)_2_, affording the corresponding thioselenation [27], thiotelluration, and selenotelluration [28] products, in good yields, respectively [29,30,31,32]. In these reactions, highly reactive chalcogeno radicals attack the terminal position of alkynes, whereas dichalcogenides bearing a higher carbon-radical-capturing ability preferentially capture the alkenyl radicals formed at the inner position of alkynes. Accordingly, the relative reactivities of chalcogeno radicals and dichalcogenides are as follows: PhS• > PhSe• > PhTe•; (PhTe)_2_ > (PhSe)_2_ > (PhS)_2_. These orders of reactivities are fully supported by the several kinetic studies reported (Scheme 1).

Since organic diselenides have an excellent carbon-radical-capturing ability, a radical addition of (PhSe)_2_ and organic peroxide such as benzoyl peroxide (BPO, (PhC(=O)O)_2_) to alkynes was attempted under thermal conditions (Equation (2)) [33,34,35,36]. Thermal decomposition of BPO generates PhC(=O)O•, which is safely trapped with (PhSe)_2_ to form PhC(=O)SePh as a key electrophilic reagent. In the case of internal alkynes, benzoyloxyselenation of the alkynes successfully proceeded, as shown in Equation (2). In the case of terminal alkynes, the benzoyloxy group was eliminated with the acetylenic proton of terminal alkynes to afford the corresponding alkynyl selenides (R-C≡C-SePh) in good yields.


(2)

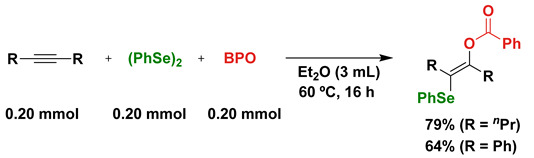



In the case of group 15–16 binary systems, a similar photoinduced radical addition to terminal alkynes was examined using binary systems of tetraphenyl diphosphine and diphenyl dichalcogenides. As can be seen from the results of Scheme 1, the desired radical addition reactions proceed successfully affording the corresponding thiophosphination [37,38], selenophosphination [39], and phosphinotelluration [40] products, respectively, with excellent regioselectivity [41,42,43,44,45].

Accordingly, the relative reactivity of the heteroatom-centered radicals is as follows: PhS• > PhSe• > Ph_2_P• > PhTe•. Although Ph_2_P• might be more reactive than PhSe•, the presence of two substituents on the phosphorus center might contribute to the relative reactivity between PhSe• and Ph_2_P•. Since there is no kinetic detail about the carbon-radical trapping ability of (Ph_2_P)_2_ and chalcogeno phosphines such as Ph_2_P–YPh (Y = S, Se, and Te), the relative reactivity of carbon-radical trapping is unclear at present.

In the case of interelement compounds including group 14 elements, the generation of radical species is difficult due to the strong bond dissociation energy. As rare examples, interelement compounds bearing a carbon–heavy-element bond such as C–I, C–Te, and C–Bi can generate the corresponding carbon radicals upon photoirradiation or in the presence of a radical initiator. For example, the combination of perfluoroalkyl iodide (R_F_I) and (PhSe)_2_ or (PhTe)_2_ leads to a regioselective perfluoroalkyl-selenation or -telluration of alkynes (Equations (3) and (4)) [46,47,48].


(3)

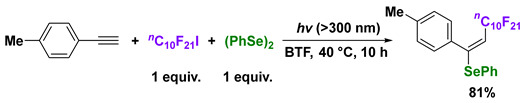




(4)

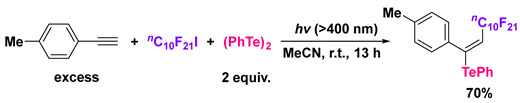



In sharp contrast, a mixed system of R_F_I and (Ph_2_P)_2_ did not work well with the radical addition to alkynes probably because (Ph_2_P)_2_ is bulkier than (PhSe)_2_. In place of the radical addition product, *P*-fluorous phosphine (R_F_PPh_2_) was obtained in almost quantitative yield [49,50,51]. The synthesized *P*-fluorous phosphine can be used as an excellent ligand for transition metal catalysts. For example, palladium catalysts having R_F_PPh_2_ as a ligand can be employed as recycling catalysts for several cross-coupling reactions such as Sonogashira coupling using a fluorous biphasic system (FBS) (Equation (5)) [52,53].


(5)

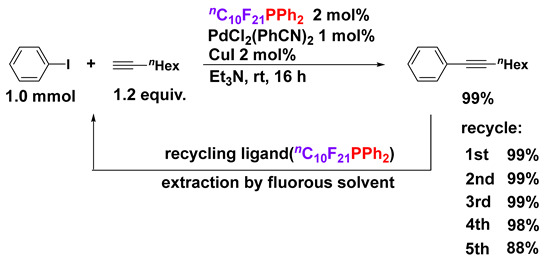




(6)

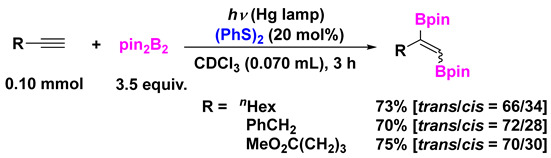



As with the group 13–16 binary system such as (Bpin)_2_ and (PhS)_2_, very interestingly, radical addition of (Bpin)_2_ to terminal alkynes proceeded successfully, despite that the radical addition did not occur in the absence of (PhS)_2_ (Equation (6)) [54,55,56,57,58,59,60].

When Ph_3_P was used as an additive, lower yields but excellent stereoselectivity were observed (e.g., 32% [91/9] (R = *^n^*Hex); 39% [91/9] (MeO_2_C(CH_2_)_3_)). Unfortunately, (PhSe)_2_ and (PhTe)_2_ were ineffective for the photoinduced diboration of alkynes. This is probably because the coordination ability of (PhSe)_2_ and (PhTe)_2_ to (Bpin)_2_ is relatively lower than that of (PhS)_2_. In the absence of additives, the formed radicals such as •B(pin) have 5-electron radical species that are unstable because they are far away from the octet. However, 7-electron radical species formed by the coordination of (PhS)_2_ or PPh_3_ are conceivably more stable.

Using a (PhS)_2_-(PhSe)_2_ binary system as a representative mixed system of interelement compounds, we next examined a photoinduced radical addition to a series of unsaturated compounds such as allenes, conjugate dienes, alkenes, vinylcyclopropanes, and isocyanides (for the detailed Scheme, see ref. [61]).

Among these unsaturated compounds, allenes exhibit the highest reactivity toward heteroatom-centered radicals. For example, (PhSe)_2_ alone cannot efficiently add to alkenes, conjugated dienes, vinylcyclopropanes, and isocyanides upon irradiation, due to the low reactivity of PhSe• with respect to ordinary unsaturated bonds. (PhSe)_2_ can photochemically add to alkynes under high concentration conditions [62]. However, the photoinduced addition of (PhSe)_2_ to allenes was complete within 1 h even under dilute conditions.

Compared to (PhS)_2_, (PhSe)_2_ has a greater absorption in the near-UV to visible region. Therefore, in the (PhS)_2_-(PhSe)_2_ binary system, (PhSe)_2_ preferentially undergoes homolysis to form PhSe• when irradiated with the light of this region. In the case of ordinary unsaturated compounds such as alkenes and alkynes, the generated PhSe• preferentially attacks (PhS)_2_ to generate PhS•, which in turn attacks alkenes and alkynes. In contrast, in the case of allenes, PhSe• preferentially reacts with allenes to give the diselenide adduct first. Upon continued photoirradiation, the diselenide adduct gradually transformed into the corresponding thioselenation product by replacing the phenylseleno group at the allylic position with a thio group [63,64].

The photoinduced thioselenation of conjugate dienes proceeded via the generation of allylic radical by the attack of the thiyl radical, followed by trapping the allylic radical with (PhSe)_2_ [65]. Similarly, the photoinduced thioselenation of vinylcyclopropanes involved the generation of cyclopropylcarbinyl radicals by the attack of the thiyl radical and the subsequent ring opening to generate the terminal carbon radical, which was captured with (PhSe)_2_ [28]. The thioselenation method could be applied to aromatic isocyanides [66,67,68,69].

In general, the photoinduced reaction of conjugate dienes in the presence of (PhS)_2_ induces polymerization of the dienes. Very interestingly, however, when the same reaction was conducted in the presence of 30 mol% of (PhSe)_2_, the polymerization was suppressed, and instead, the dithiolation products were obtained selectively (Equation (7)) [65].


(7)

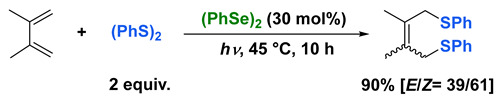



The present (PhSe)_2_-assisted dithiolation could be applied to the dithiolation of aliphatic isocyanides. Moreover, the photoinduced dithiolation of allenes could be attained by the coexistence of 30 mol% of (PhTe)_2_ [63].

## 4. Photoinduced Radical Addition of Interelement Compounds to Several Unsaturated Compounds

Based on the insight into the efficacy of interelement compounds bearing excellent carbon-radical-capturing ability and the relative reactivity of a series of unsaturated compounds, we next demonstrated the multicomponent reactions using two or more unsaturated compounds. As discussed in Section 3, the relative reactivity of unsaturated compounds toward heteroatom-centered radicals is shown as follows: (highly reactive) allene > alkyne > diene > alkene (less reactive). In addition, the order of the carbon-radical-capturing ability is as follows: (PhTe)_2_ > (PhSe)_2_ > (PhS)_2_. In the case of the low carbon-radical-capturing ability such as (PhS)_2_, radical polymerization is also expected to proceed. Although (PhTe)_2_ has an excellent carbon-radical-capturing ability, the weaker bond energy of C–Te (47.8 kcal/mol) compared with those of C–S and C–Se (65.1 and 56.0 kcal/mol, respectively) might induce the living polymerization of unsaturated compounds. Accordingly, (PhSe)_2_ is the best choice of mediator for the desired sequential addition to muticomponent unsaturated compounds.

To attain such muticomponent radical reactions, it is of great importance to consider the stability of carbon radical species, which can be estimated by comparing the bond dissociation energy of a series of C–H bonds: 105 kcal/mol (CH_3_–H); 101 kcal/mol (RCH_2_–H, *primary*); 98.5 kcal/mol (R_2_CH–H, *secondary*); 96.5 kcal/mol (R_3_C–H, *tertiary*); 87 kcal/mol (RCH=CHCH(R)–H, *allylic*); 112 kcal/mol (Ph–H, *phenyl* (*or arylic*)); 87 kcal/mol (PhCH_2_–H, *benzylic*). As can be seen from these data, aryl radicals (Ar•) are the most powerful radical species, whereas allylic or benzylic radicals are relatively stable carbon radicals, in which it is difficult to induce further addition to usual unsaturated compounds intermolecularly.

Next, we need to consider which unsaturated compounds are suitable for multicomponent radical addition of interelement compounds. In the case of allenes, powerful heteroatom-centered radicals such as PhS• attack both terminal and internal positions of allenes, resulting in poor regioselectivity (central attack/internal attack = 75/25). In contrast, less reactive heteroatom-centered radicals such as PhSe• and PhTe• predominantly attack the central carbon of allenes, generating allylic radicals that are difficult to add to other unsaturated compounds. Allenes are therefore not suitable candidates for unsaturated compounds attacked first by heteroatom-centered radicals in multicomponent radical addition reactions.

**Scheme 2:**
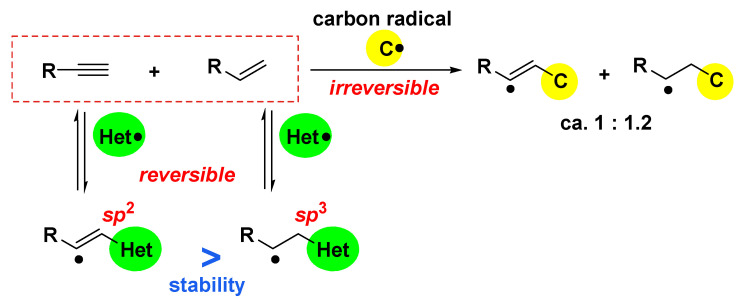
Considerations for the selection of unsaturated compounds for multicomponent reactions.

When a mixture of alkyne and alkene is employed in multicomponent radical addition reactions, carbon radicals irreversibly add to both alkyne and alkene with an alkene/alkyne ratio of ca. 1.2 (Scheme 2). In the case of heteroatom-centered radicals, however, the addition proceeds reversibly, preferring the more stable β-heteroatom-substituted alkenyl radicals over β-heteroatom-substituted alkyl radicals. This is most probably because the dissociation energy of the *sp*^2^ carbon-heteroatom bond is stronger than that of the *sp*^3^ carbon-heteroatom bond. Based on these considerations, the reaction of heteroatom-centered radicals with a mixture of alkyne and alkene might preferentially generate β-heteroatom-substituted alkenyl radicals as a key species for the multicomponent reactions.

**Scheme 3:**
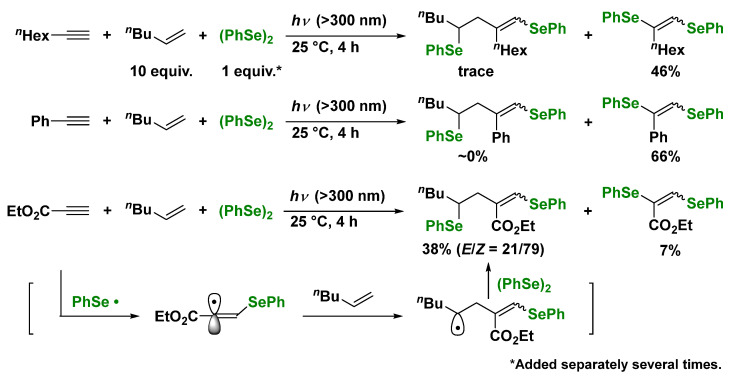
Diselenide-mediated addition to 1-hexene using several alkynes.

**Scheme 4:**
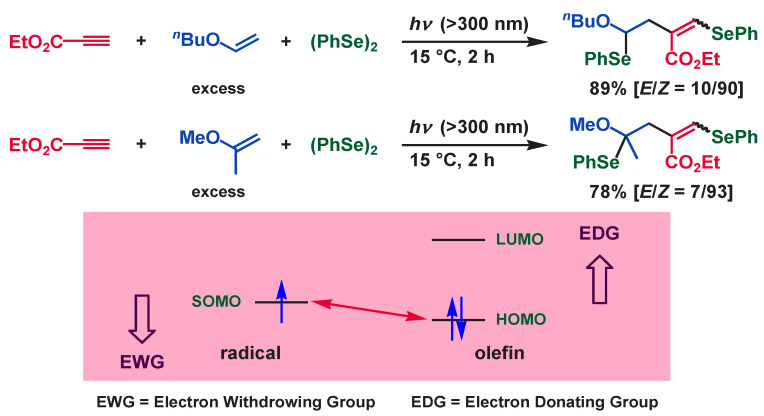
Diselenide-mediated sequential addition to ethyl propiolate and vinyl ethers.

Thus, we examined the photoinduced sequential addition of (PhSe)_2_ to 1-hexene using several alkynes (Scheme 3). Aliphatic and aromatic alkynes such as 1-octyne and phenylacetylene, which generate vinylic σ- and π-radicals, respectively, were both ineffective in the sequential addition with 1-hexene, and the diselenide adducts to the alkynes were mainly obtained. However, when an electron-deficient alkyne such as ethyl propiolate was employed, the desired sequential addition product was obtained with a decrease in the formation of the diselenide adduct. The sequential addition might proceed via the selective attack of the seleno radical to ethyl propiolate, followed by the addition of the formed vinyl radical to 1-hexene. Then, the formed alkyl radical is captured with (PhSe)_2_. The electron-withdrawing group might prolong the lifetime of the vinyl radical species to further react with alkenes. This can be explained by the orbital interaction, as shown in Scheme 4 [70].

The singly occupied molecular orbital (SOMO) of radical species can be stabilized by the electron-withdrawing group and close to the highest occupied molecular orbital (HOMO) of alkene. To emphasize the SOMO-HOMO interaction, the electron-donating group is introduced into the alkene.

Based on this concept, we attempted the photoinduced reaction of ethyl propiolate with vinylic ethers such as butyl vinyl ether and methyl 2-propenyl ether, which successfully afforded the sequential addition products in high yields upon shorter photoirradiation (Scheme 4).

Furthermore, photoinduced sequential addition of (PhSe)_2_ to ethyl propiolate and conjugate diene or vinylcyclopropane was also examined. Even using excess amounts of conjugate diene or vinylcyclopropane, PhSe• selectively attacked ethyl propiolate to generate the corresponding vinylic radical that was sequentially added to conjugate diene or vinylcyclopropane. The resulting allylic radical or primary carbon radical formed by the ring opening of the cyclopropane was captured by (PhSe)_2_ to afford the corresponding multicomponent coupling products in good yields (Equations (8) and (9)) [70,71].


(8)

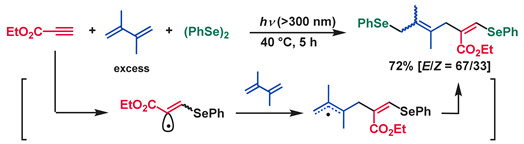




(9)

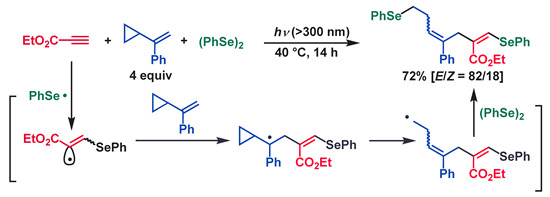



**Scheme 5:**
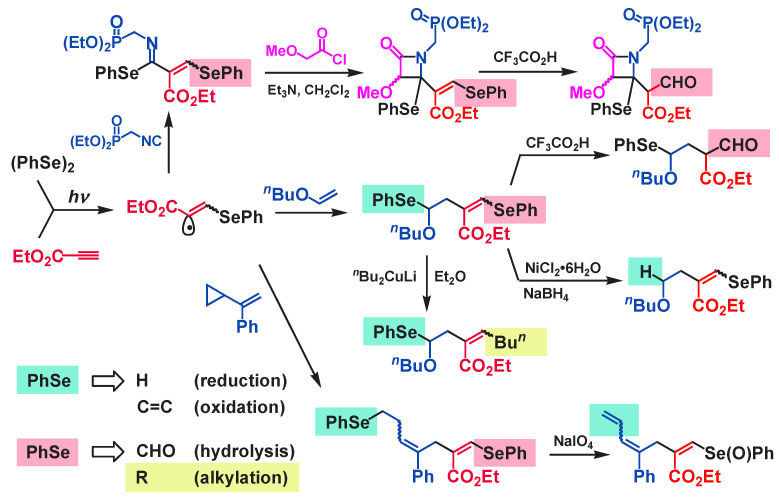
Synthetic utility of multicomponent coupling products bearing seleno groups.

When excess amounts of isocyanides are used for this sequential addition of (PhSe)_2_ to ethyl or methyl propiolate, the generated vinyl radicals are trapped with isocyanides to afford the corresponding α,β-unsaturated imine derivatives in good yields (Equation (10)) [72]. As can be seen from Scheme 5, in the case of isocyanide having (EtO)_2_P(O)-group, a similar imine derivative was formed in 58% yield. The following reaction of this imine derivative with the in situ generated ketene (MeO–CH=C=O) successfully led to the formation of a β-lactam derivative (85% yield), the hydrolysis of which afforded the corresponding β-lactam derivative having formyl group (51% yield). This β-lactam derivative is a key intermediate for the synthesis of carbapenem analogs by conducting a Wittig-type reaction [72].


(10)

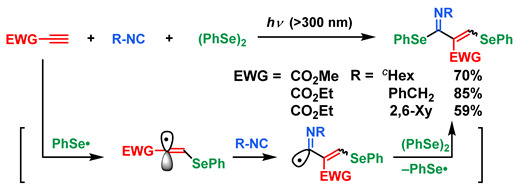



Scheme 5 also indicates the synthetic utility of a series of multicomponent coupling products bearing seleno groups. A vinylic seleno group can be converted into not only a formyl group but also alkyl groups by the reaction with organocuprates (e.g., *^n^*Bu_2_CuLi, Et_2_O, −110 °C~r.t., 1 h, 84% [*E*/*Z* = 7/93] from the vinyl selenide [*E*/*Z* = 10/90]) [70]. On the other hand, alkyl selenides can lead to the corresponding alkenes by selenoxide *syn*-elimination or can be removed by reduction with NiCl_2_/NaBH_4_.

## 5. Photoinduced Multicomponent Reactions of Interelement Compounds Involving Cyclization Process

**Scheme 6:**
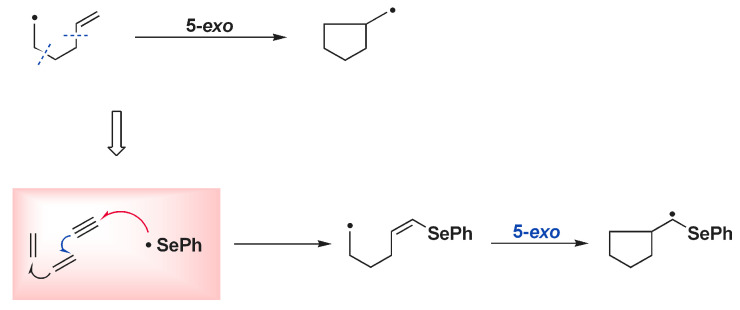
Strategy for multicomponent reaction incorporating radical cyclization.

In general, intramolecular reactions are 1000 times faster than intermolecular reactions. Therefore, by incorporating intramolecular reactions into the radical addition reactions of interelement compounds, it is expected that more advanced multicomponent reactions can be designed. 5-*Exo* cyclization is one of the most important intramolecular radical reactions. For example, 5-hexenyl radical undergoes 5-*exo* cyclization to generate cyclopentylmethyl radical (*k* = 2.3 × 10^5^ s^−1^). Considering the sequential addition to an alkyne and alkene by a seleno radical, a 5-hexenyl radical might be synthesized by the sequential addition to an alkyne and two molecules of alkenes (Scheme 6). As mentioned in Section 4, the seleno radical first attacks ethyl propionate and then the electron-rich alkene, producing a carbon radical with an alkoxy group at the α-position. Now then, what kind of alkene is desirable as the next alkene to be attacked by this carbon radical? It is expected that the SOMO energy level of the carbon radicals with an alkoxy group at the α-position is higher. If the LUMO of an alkene interacts with this SOMO, it would be preferable to use an alkene with an electron-withdrawing group which lowers the energy level of the LUMO.

**Scheme 7:**
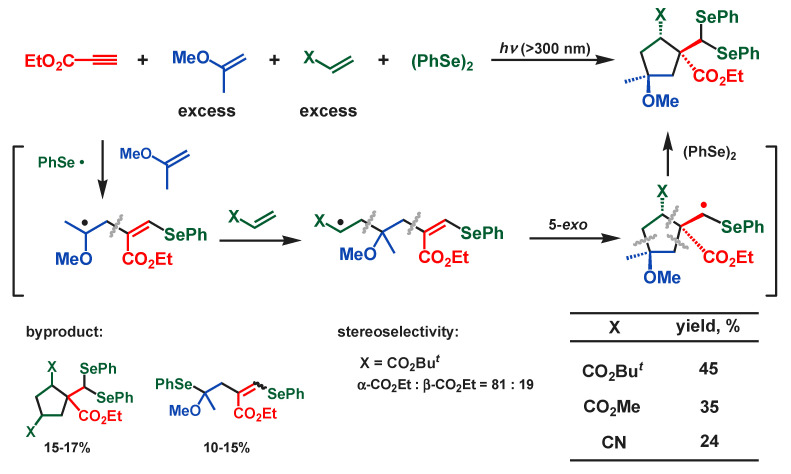
(PhSe)_2_–induced multicomponent reaction via radical cyclization.

Thus, we examined the photoinduced reaction of (PhSe)_2_ with ethyl propiolate, methyl 2-propenyl ether, and acrylate derivatives or acrylonitrile (Scheme 7). The incorporation of 5-*exo* cyclization prevented further addition (or oligomerization), providing the corresponding cyclic diselenides that were obtained in moderate yields [73,74,75,76,77,78,79,80,81,82,83].

In this reaction, interestingly, five-membered cyclization products incorporating two electron-poor alkenes were also formed as byproducts in 15–17% yields. This means the carbon radical intermediates bearing electron-withdrawing groups are stable enough to induce the sequential addition of two molecules of the alkenes, and in addition, the presence of the 5-*exo* cyclization process can inhibit further addition (or oligomerization), resulting in the formation of the corresponding five-membered cyclization product as a stereoisomeric mixture (Equation (11)).


(11)

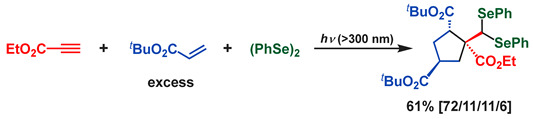



**Scheme 8:**
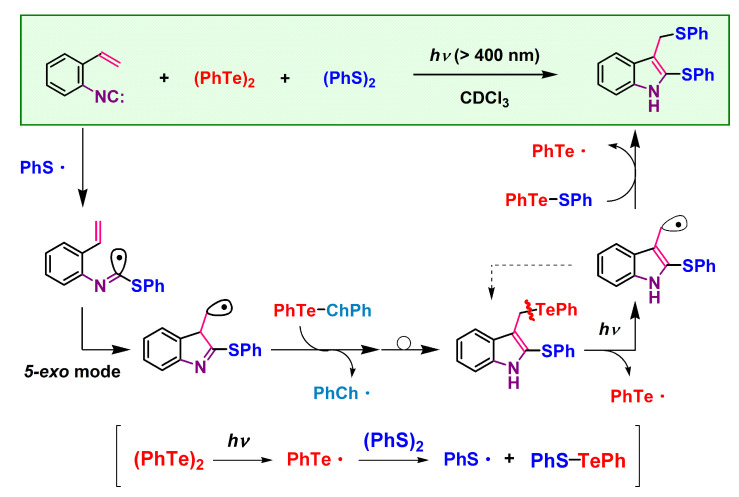
(PhTe)_2_-mediated dithiolative cyclization of *o*-alkenylphenyl isocyanide.

When a mixture of *N*-aryl isocyanides having an *o*-alkenyl group, (PhS)_2_, and (PhTe)_2_ was irradiated with visible light, a novel dithiolation incorporating 5-*exo*-type cyclization proceeded successfully to afford the corresponding 2-(phenylthiol)-3-(phenylthiomethyl)indene derivatives in good yields (Scheme 8). In this reaction, PhTe• formed by the visible light irradiation attacks (PhS)_2_ to generate PhS•, which adds to the isocyano group producing the imidoyl radical. In general, photoinduced thiotelluration of PhNC is difficult to take place due to the instability of the thiotelluartion product under the photoirradiation condition. Surprisingly, however, the 5-*exo*-type cyclization could induce the radical addition of (PhS)_2_ to the aryl isocyanide, providing the indene disulfide by trapping the indenylmethyl radical with PhSTePh [84,85,86,87,88,89,90,91,92,93,94,95,96].

**Scheme 9:**
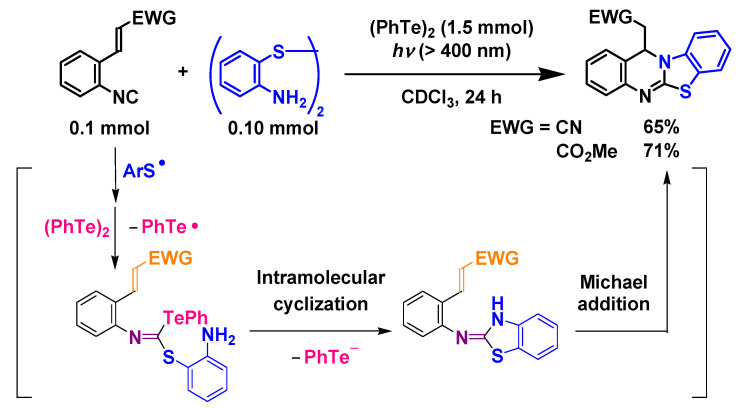
(PhTe)_2_-mediated synthesis of multi-cyclic compounds from *o*-alkenylphenyl isocyanide.

Furthermore, when bis(*o*-aminophenyl) disulfide was used for the (PhTe)_2_-mediated reaction with *o*-alkenylphenyl isocyanide having an electron-withdrawing group at the terminal position of the *o*-alkenyl group, multicyclic compounds could be synthesized via ionic intramolecular cyclization processes (Scheme 9).

Thiyl radical attacks the isocyano group to generate an imidoyl radical, which is selectively trapped with (PhTe)_2_. Since the telluro group introduced is a good leaving group, the *o*-amino group induces nucleophilic substitution intramolecularly. Furthermore, Michael-type addition occurs between the amino group and the alkenyl group to directly construct a tetracyclic ring system. This one-pot sequential reaction is very interesting because ionic intramolecular cyclization reactions can be incorporated into a multicomponent radical reaction system.

## 6. Conclusions

This mini-review focuses on multicomponent reactions between unsaturated compounds and mutual component compounds by radical addition and cyclization reactions. Radical reactions have high tolerance to functional groups and low sensitivity to solvents, making them very suitable for designing multicomponent reactions [97,98,99,100,101,102,103,104,105,106,107,108,109,110,111,112,113,114,115,116,117,118,119,120,121,122,123,124,125,126,127,128,129,130,131,132,133].

To construct highly selective multicomponent radical reactions, it is important to consider the following: (1) the relative stability of carbon radicals based on the bond dissociation energy; (2) orbital interactions between SOMO and HOMO or LUMO; (3) kinetic data on addition, cyclization, etc.; (4) the characteristics of heteroatoms, especially upon photoirradiation; (5) the relative reactivity of heteroatom-centered radicals; (6) the relative carbon-radical-capturing ability of interelement compounds; (7) the control of the reaction by the optimization of the substrate concentration; (8) the use of heteroatom mixed reaction systems; and (9) the use of intramolecular reactions.

We hope that this mini-review will help researchers develop new multicomponent reactions in an environmentally friendly manner.
